# Dietary silver nanoparticles as immunostimulant on rohu (*Labeo rohita*): Effects on the growth, cellular ultrastructure, immune-gene expression, and survival against *Aeromonas hydrophila*

**DOI:** 10.1016/j.fsirep.2022.100080

**Published:** 2022-12-29

**Authors:** Omoniyi Michael Popoola, Bijay Kumar Behera, Vikash Kumar

**Affiliations:** a Department of Fisheries and Aquaculture Technology, Federal University of Technology, Akure, Nigeria; b ICAR-Central Inland Fisheries Research Institute, Barrackpore, Kolkata, West Bengal 700 120, India

**Keywords:** Labeo rohita, Immunostimulants, Silver nanoparticles, Aeromonas hydrophila, Immune genes

## Abstract

The current study aimed at assessing the immunostimulatory properties of silver nanoparticles (AgNPs) on *Labeo rohita*, and understanding how it affects the growth, cellular ultrastructure, the expression level of immune genes, and infection risk from *Aeromonas hydrophila*. Fish (avg wt: 30.1±3.26 g) were fed diets with four separate AgNP inclusion levels (0 µgKg^−1^ [basic diet, T1], 10 µgKg^−1^ [T2], 15 µgKg^−1^ [T3], and 20 µgKg^−1^ [T4]) for 56 days. After the feeding trial, growth, histological, immunological parameters, and protective immune response against *A. hydrophila* were assessed. The fish in the treatment groups including T1(control), the T3 growth indices, such as specific growth rate (7.56±0.26) and percent weight gain (231.05±3.21), was statistically higher (P < 0.05). In the immunological and oxidative parameters, levels of SOD and catalase decreased in correlation with a rise in the inclusion doses of AgNP in the liver, and a reduction in catalase values was recorded in the gill. With the addition of AgNP, the NBT value was decreased in the gill, and T3 had a considerably larger (P<0.05) value in the liver (0.493±0.02). The kidney of the *L. rohita* fed AgNP (0 and20 µgKg^−1^ AgNP) showed expansion through Bowman's gaps, severing of glomeruli with haemorrhage, as well as atrophic spots between its gaps. The liver showed fibrosis, karyolysis, and the removal of the hepatocytes wall. The gill, liver, kidney, and muscle of fish-fed diets supplemented with AgNP, showed that interleukin-8 (IL-8), and cyclooxygenase-2 (COX-2), were up-regulated. Expression was considerably higher in T3 compared with the control. However, the control group that wasn't given AgNP supplemented diet had increased levels of TGF-beta. Additionally, fish on the T3 diet showed much greater post-challenge survival rates (90%). These findings strongly suggest that dietary inclusion of AgNP (at 10 and 15 µgKg^−1^ feed) enhances growth, health, and protective immune response against *A. hydrophila*.

## Highlights

• Silver nanoparticles (AgNPs) can stimulate immune modulation.
• Silver nanoparticles (AgNPs) can effectively improve growth and survival against *A. hydrophila* in *L rohita*.
• AgNPs can be used as immunostimulants and for growth enhancement in cultured fish.

## 1 Introduction

Many essential micronutrients that the human body need, including proteins, vitamins, and minerals, can be found in fish. Due to population growth and the resulting rapid decline in fish catches from rivers, seas, and oceans, there is a high demand for fish as a cost-effective source of complete protein. However, the global aquaculture industry is gradually expanding due to the availability of necessary technologies [1], unfortunately, poor methods of fish cultivation have led to numerous fish infections [2].

A significant barrier to the expanding aquaculture industry and a significant financial drain is infection. Due to their shortcomings several infection management techniques, like the administration of antibiotics or vaccinations, are ineffective [3]. Although antimicrobial agents and therapeutic medications are used in aquaculture systems for preventative and therapeutic purposes, their negative effects have been hotly debated. As a result, using immunostimulants to prevent disease has gained more attention [4].

Numerous immunostimulants have been intensively investigated in a variety of fish species, including lipopolysaccharide, glucans, peptidoglycan, vitamin C, and others. AgNP is used in agriculture and medicine because of its distinctive properties, which allow for novel applications like diagnostics. It can also be mixed with other metals or chemicals to generate new materials with improved performance [5].

AgNP and other nanoparticles have received much interest in the area of fish farming as a specific method of treating various bacterial, fungal, and viral illnesses due to the rising challenges regarding bacteria that are resistant to antibiotics [6]. The bactericidal action of AgNP was found to be effective against *Aeromonas* sp, which is believed to be the most prevalent strain of pathogenic bacteria threatening the success of the aquaculture business [6,7]. In many aquatic species, AgNP is effective against *A. hydrophila* [8]. Moreover, dietary AgNP applied rightly could reduce biological and chemical stress while improving zootechnical performance, immunological health, and aquatic animal longevity [9]. According to Britto [10], the water-borne bacteria *A. hydrophila* can cause fish to develop red sores, ulcers, and septicemia, especially *L. rohita* and causes diarrhea in immunocompromised individuals [11].

The most commercially important freshwater-farmed fish species is rohu (Labeo rohita), which is produced in India, one of the top three producers of freshwater fish worldwide [12]. This species is benthopelagic and extremely susceptible to maladies, particularly diseases like ulcerative, fin, and tail rot, which can be spread to other fish species that are in the same habitat [13].

Fish are immunoprimitive animals that mostly rely on innate immunological responses, which can be stimulated by immunostimulants, to protect them from disease. The utility of AgNP as an immunological stimulant in *L. rohita* has not been thoroughly investigated. Both the fish's immune system and capacity to generate better-quality fish from varied and intense fish farming will be strengthened by using it as an emerging immunostimulant for *L rohita*. Additionally, prior research has shown that *A. hydrophila* is highly susceptible to developing antibiotic resistance [14].

Thus, the goal of the study is to assess AgNP's capacity to stimulate the immune system in *L. rohita* by assessing its effects of supplementation on growth, the histology of some immunoprotective organs, and the expression of particular genes associated with immunity.

## 2 Material and methods

The research was carried out in accordance with the Guide for the Use of Experimental Animals of the ICAR-Central Inland Fisheries Research Institute, Barrackpore, India.

### 2.1 Silver nanoparticle preparation and characterization

In the Argovit, about 12 mg/ml of metallic silver was allowed to stabilise to 188 mg/ml polyvinylpyrrolidone (PVP). Argovit is simply a solution of scattered silver nanoparticles (AgNP) coated with PVP at a 200 mg/ml concentration in water (20%). Based on the quantity of silver present in the Argovit preparation, AgNP dilution was estimated. Without any additional additives, samples were made through RPMI-1640 media and stored at 4°C in dark conditions. Based on the findings of HRTEM with a microscope (JEOL-JEM-2010), the size distribution and morphology of AgNP were determined. Employing a green laser operating at 1/4 532 nm at 25°C, a DLS Zetasizer Nano NS (DTS 1060, UK) was utilised to measure both the zeta potential and the hydrodynamic radius. AgNP was examined with spectroscopy (UV-Vis) within the 200-900 nm range (Agilent Technologies, USA). With the help of a universal diamond ATR top plate attachment (PerkinElmer, USA), Further characteristics of lyophilized Argovit were done by FTIR-ATR analysis with a resolution of 2 cm^−1^ in the 400–4000 cm^−1^ range. The sample's spectra were compared to that of regular solid PVP (Mw: 100kD).

### 2.2 Experimental design with diet formulation

From a local fish farmer in Kolkata, India, 180 juveniles of *L. rohita* (avg wt: 30.1±3.26 g) were bought. The fish were acclimatized for 15 days before the experiment started. The fish (20 pieces/tank) were randomly distributed into 12 circular tanks, each with a capacity of 80 litres in the experimental units of ICAR-Central Inland Fisheries Research Institute, Barrrackpore, India. The tanks were provided with aeration and optimum water quality parameters including pH (7.8 ± 0.15), dissolved oxygen levels (5.6 mgL^−1^), water temperature (28.0±0.3°C), ammonia, nitrite, and nitrate (0.09 mgL^−1^), were maintained throughout the experiment.

For 56 days, three percent of the body weight of the prepared diets were given at various AgNP levels (0, 10, 15, and 20 µg kg-1) (Table 1). Only a control diet was given to the test fish before and after the 56-day feeding trial.

**Table 1:** Experimental diet composition with varying inclusion level of AgNP

s/no	Composition	% inclusion	% inclusion	% inclusion	% inclusion	Proximate composition	
1	Soybean cake	36.0	36.0	36.0	36.0	Protein	31 ± 0.10
2	Mustard cake	45.50	45.50	45.50	45.50	Lipid	10.2± 1.31
3	Deoiled rice bran	2.0	2.0	2.0	2.0	Moisture Ash	9.2± 0.41
4	Fish meal	5.0	5.0	5.0	5.0	Ash	10.1± 0.85
5	Oil mix	1.5	1.5	1.5	1.5		
6	Vitamin premix	1.0	1.0	1.0	1.0		
7	Mineral mixture	1.0	1.0	1.0	1.0		
8	Herbal attractant	2.0	2.0	2.0	2.0		
9	Probiotics	2.0	2.0	2.0	2.0		
10	Cysteine	0.25	0.25	0.25	0.25		
11	Methionine	0.25	0.25	0.25	0.25		
12	Tryptophan	1.5	1.5	1.5	1.5		
13	Antox	1.0	1.0	1.0	1.0		
14	Aquace	1.0	1.0	1.0	1.0		
	AgNP	0	10	15	20		

### 2.3 Feeding efficiency and growth performance indicators

The following equations were used to determine the protein efficiency ratio (PER), average weight growth (AWG), percentage weight gain (PWG), specific growth rate (SGR), and feed conversion ratio (FCR) based on the pool weight and the number of fish in each tank:

$$FCR=\frac{\text{feed}\;\text{given}\;\text{to}\;\text{the}\;\text{fish}\;\left(\text{dry}\;\text{weight}\right)}{\text{fish}\;\text{weight}\;\text{gain}}$$

AWG=finalaverageweightgain−theinitialaverageweightofthefish

$$PWG\;\left(\%\right)=\frac{\text{MWG}}{\text{initial}\;\text{average}\;\text{weight}\;\text{of}\;\text{fish}}x100$$

$$SGR=\frac{100\;\text{ln}\;\left[\text{final}\;\text{weight}\;-\text{initial}\;\text{weight}\right]}{\text{total}\;\text{trial}\;\text{period}}$$

$$\mathrm{SR}\;\left(\%\right)=\frac{\text{total}\;\text{fish}\;\text{count}\;\text{after}\;\text{the}\;\text{trial}}{\text{initial}\;\text{number}\;\text{of}\;\text{fish}\;\text{stocked}\;\text{at}\;\text{the}\;\text{beginning}}x100$$

### 2.4 Sampled collection

Randomly chosen fish were sacrificed from each tank (5 fish /tank, 15 fish total for each treatment) at the end of the trial. Samples, including kidney, muscle, and gill, were preserved in NBF for histological analysis, while another set, including liver, kidney, and gill collected for analysis related to oxidative stress (preserved in 2% sucrose solution). Additionally, for gene expression analysis, liver, muscle, kidney, and gill samples were directly kept in RNA Later (Qiagen, USA) at -80 °C.

### 2.5 Pathogen source, preparation, and challenge assay

In this study, *A. hydrophila* (MG754418), a strain of highly pathogenic bacteria identified by the ICAR - CIFRI Barrackpore, India was used. 100 ml capacity conical flask filled with autoclaved 30 ml Tryptic Soy Broth (TSB; Merck) was used in the culture of the bacterium as it developed to a log phase. Centrifugation was performed on the bacterial culture for 20 minutes at 3500 x g and 4°C to collect the bacteria pellets. Thereafter, a sterile 0.15 M phosphate-buffered solution (PBS) (pH 7.2) was used to clean the bacterial pellets. Lethal dose 50% (LD_50_) trial results were used to re-dissolve the pellets in PBS and adjust the concentration to 1.5 × 10^8^ cfuml^−1^ [15]. Following injection (15 pieces/treatment, 5 fish/tank), the fish were closely observed for 15 days, and pathological changes and mortalities were noted. Dead and morbid fish were gathered after challenged in confirming *A. hydrophila* as the fatality's cause, and the challenging strain was aseptically isolated and recognised from various tissue samples (intestine, muscle, liver, kidneys, gills, and eyes).

### 2.6 Antioxidants, and immune-related parameters

Using the TissueLyser II, the liver and gill samples from the test and control fish were homogenised in sucrose solution (0.25 M) (Qiagen, Hilden, Germany). For the enzyme test, the clear upper liquid was then put into sterile 2 ml tubes and refrigerated at -40°C. For SOD activity (pH 10.2), a reaction mixture containing bicarbonate buffer (0.1M, pH 10.2) was combined with the samples’ homogenate and immediately added epinephrine 100 µl to the mixture. The OD was recorded at 480 nm every 30 seconds. The catalase activity was evaluated utilising Caliborne [16] method using phosphate buffer (50 mM, pH 7.2) with 50 mM H_2_O_2_ and OD was recorded at 240 nm. To measure the respiratory burst activity, 100 µl of homogenized fish tissues (liver and gill) from each treatment were added to 0.1 ml of NBT (0.2 percent) (Sigma, USA) and then incubated at 25°C for 30 minutes. After the aforementioned combination had been incubated, about 50 µl of it was taken per sample and 1000 l of N, N-diethyl methyl formamide (Qualigens, India) was added, and the mixture was centrifuged for 5 minutes at 6,000 x g and the OD of the reaction was recorded at 540 nm.

### 2.7 Histopathology

The selected organs (gill, muscle, liver, and kidney) from *L. rohita* were taken from each treatment group, cleaned in a sterile solution, and then kept in 10% formalin buffer (NBS) for 24 h. The control group's dead fish were quickly dissected to collect the organs for analysis. The tissues were first fixed, then dehydrated in a sequence of alcohol solutions of different ratios (70, 70, 90, and 100%), immersed in paraffin, sectioned at 5 mm, and smeared in hematoxylin-eosin (H&E) [17]. A Magnus MLXi Plus 10 microscope and a 100x optical lens were used to take microscopic pictures of the slides.

### 2.8 Gene expression analysis and RNA isolation

To assess the expression of the cytokines transforming growth factor (TGF-β), interleukin (IL-8), and cyclooxygenase-2, (COX-2) samples of the liver, gills, kidney, and muscle collected and earlier preserved in RNA Later were used. The TRIzol technique was used to extract RNA from the obtained tissues. Subsequently, the quantity and quality of the extracted RNA were measured Spectrophotometrically with NanoDropTM ND-1000 (Thermo Fisher Scientific, USA).

To measure the mRNA expression folds of the examined genes (Table 2), RT-PCR (BIORAD) was carried out using QuantiTect SYBR Green RT-PCR Kit. With beta-actin (β actin) serving as a housekeeping gene, all the investigated genes were compared. The 2^−ΔΔCT^ approach [18,19] was used to determine the relative mRNA expression folds.

**Table 2:** rRT-PCR primer sequences used to analyse the expression of the target gene.

Gene of interest	Primer sequence (5′-3′)	Source
COX-2	F- ATCCTTACTCACTACAAAGG R-GCTGGTCCTTTCATGAAGTC	[20]
IL-8	F-CTGTGAAGGCATGGGTGTG R-ATCACTTTCTTCACCCAGGG	[21]
TGF-β	F-ACGCTTTATTCCCAACCAAAC R-GAAATCCTTGCTCTGCCTCA	AF136947
β-actin	F- GACTTCGAGCAGGAGATGG R- CAAGAAGGATGGCTGGAACA	[22]

COX:  cyclooxygenase, IL: interleukin, TGF: Transforming growth factor,

### 2.9 Data analysis

**Fig. 1:**
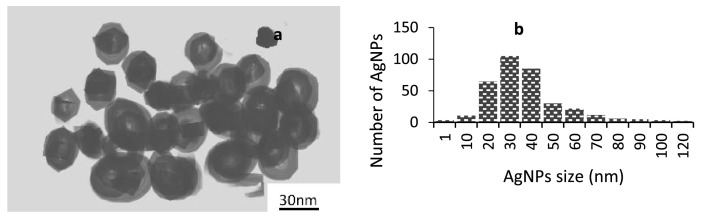
AgNP characterization: (a) TEM image of AgNP at various magnifications, (b) AgNP size distribution.

Using a one-factor analysis approach (ANOVA), the statistical analysis was done in SPSS Version 22. To separate differences between treatment means, New Duncan's Multiple Range Test (NDMRT) was used for all statistical calculations. The statistics were displayed as mean ± SD with α= 95%; P < 0.05. Fig 1

## 3 Results

### 3.1 Growth studies

**Fig. 2:**
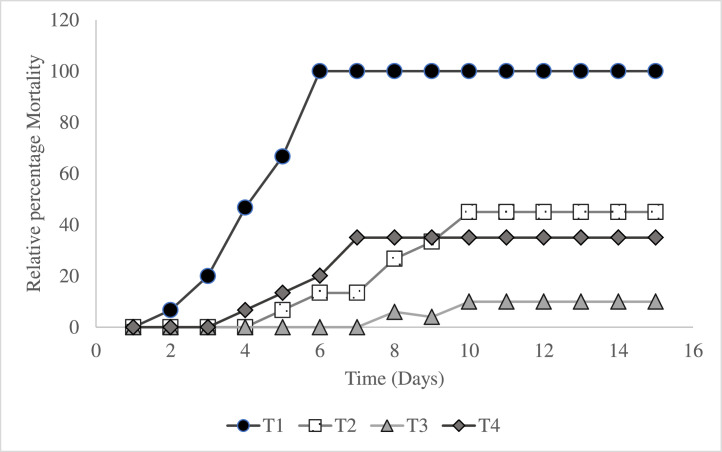
Relative percentage mortality of L. rohita against.A. hydrophila L. rohita after being fed with dietary silver nanoparticles (T1:0 µgKg−1, T2:10 µgKg−1, T3:15 µgKg−1, and T4: 20 µgKg−1 AgNP).

The performance in growth of *L. rohita* was influenced by AgNP, as shown in Table 3. Upon completion of the trial, the AgNP-included group exhibited a greater proportion of weight gain (PWG) in comparison with the control group (P < 0.05). SGR exhibited a significant variance (P < 0.05) among the different treatments, with T3 having the greatest value, in contrast to FCR, which, when compared to other treatments, was statistically higher (P < 0.05) in the control treatment. The relative survival rate does not follow a specific order and the highest percentage recorded was in T3 and nil in the control treatment (Fig. 2)

**Table 3:** Growth and survival of *Labeo rohita* given various levels of AgNP.

Growth indices	T_1_	T_2_	T_3_	T_4_
Initial weight (g)	29.98±0.01^a^	29.92±0.02^a^	31.18±0.03^a^	30.00±0.05^a^
Final Weight Gain (g)	84.37±0.31^d^	97.12±0.29^b^	99.49±0.34^a^	91.89±0.29^c^
Weight gain (WG)	54.39	67.20	68.31	61.89
WG (%)	181.42^d^	224.60^b^	219.08^a^	206.30^c^
Feed Conversion Ratio (FCR)	1.85^a^	1.50^c^	1.48^c^	1.63^b^
Specific Growth Rate (SGR)	7.14^c^	7.51^a^	7.54^a^	7.37^b^

The Data are displayed as mean ± SD (n=45). The mean with opposing letters is statistically different (P < 0. 05). T_1_:0 µgKg^−1^, T_2_:10 µgKg^−1^, T_3_:15 µgKg^−1^ and T_4_: 20 µgKg^−1^ AgNP

### 3.2 Antioxidant and immune defense-related parameters

**Fig. 3:**
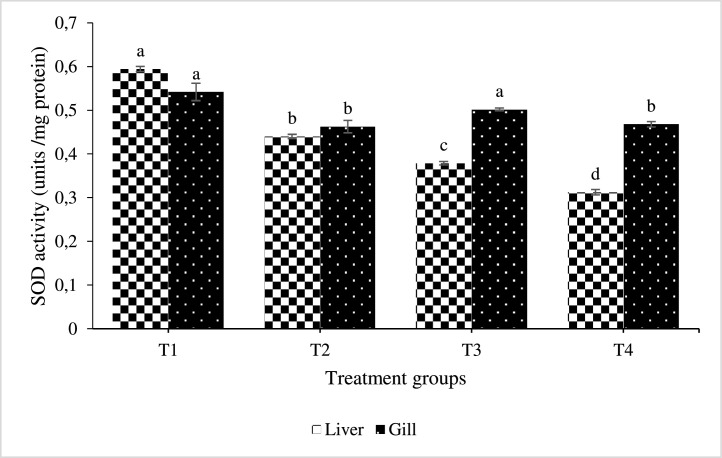
Effect of dietary silver nanoparticles on the SOD activity of L. rohita against. hydrophila. L. rohita displayed elevated superoxide dismutase activity (SOD) (AgNP) gills. However, lower values were recorded in the T1 group. The significance level is 95%; P < 0.05, which shows a notable distinction between the mean values with contrasting superscripts. (T1:0 µgKg−1, T2:10 µgKg−1, T3:15 µgKg−1 and T4: 20 µgKg−1 AgNP).

**Fig. 4:**
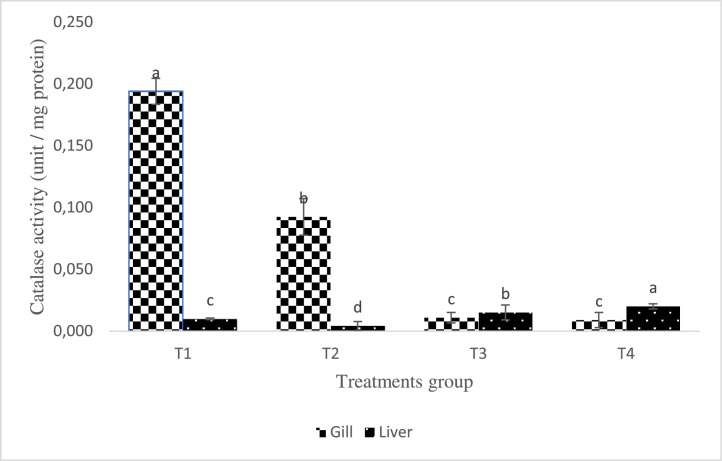
Effect of dietary silver nanoparticles on the Catalase activity of L. rohita against A. hydrophila. L. rohita displayed the decreased activity of catalase (AgNP) gills. However, the values show an increase from the T1 to T2 groups. The significance level is 95%; P < 0.05, which shows a notable distinction between the mean values with contrasting superscripts. (T1:0 µgKg−1, T2:10 µgKg−1, T3:15 µgKg−1 and T4: 20 µgKg−1 AgNP).

**Fig. 5:**
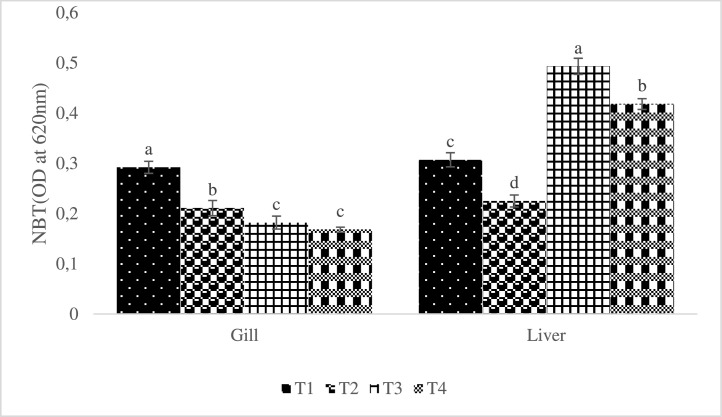
Respiratory burst activity (NBT) of Labeo rohita exposed to A. hydrophila and fed varying amounts of dietary silver nanoparticles (AgNP). (T1:0 µgKg−1, T2:10 µgKg−1, T3:15 µgKg−1 and T4: 20 µgKg−1 AgNP). The significance level is 95%; P < 0.05, which shows a notable distinction between the mean values with contrasting superscripts.

All AgNP-enriched treatment groups showed different levels (P< 0.05) of SOD and CAT activity. The SOD and CAT levels declined with increasing levels of AgNP inclusion in the treated fish's liver and gill, respectively (Fig. 3). In all groups supplemented with AgNP, superoxide dismutase activity was observed to decline in the gill in a dose-response pattern. Additionally, the group raised with AgNP included diets, in particular at higher levels, demonstrated significantly increased catalase activities (P < 0.05), though less compared with the control (Fig. 4). As opposed to that, as the amount of supplemental AgNP increased, NBT activities in all of the experimental groups decreased, with the greatest reduction occurring in T3 in the treated fish's liver and gill (Fig. 5).

### 3.3 Histopathological examination

**Fig. 6:**
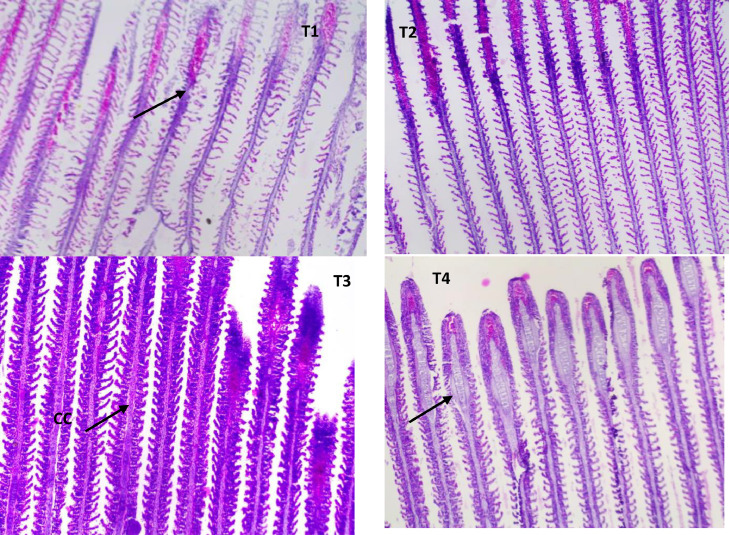
AgNP fed to and challenged by A. hydrophila, resulting in histopathological changes in the gills of L. rohita (T1:0 µgKg−1, T2:10 µgKg−1, T3:15 µgKg−1, and T4: 20 µgKg−1 AgNP). Cellular infiltrations of the epithelium (arrow) chloride cells (CC) (H and E stain, x100).

**Fig. 7:**
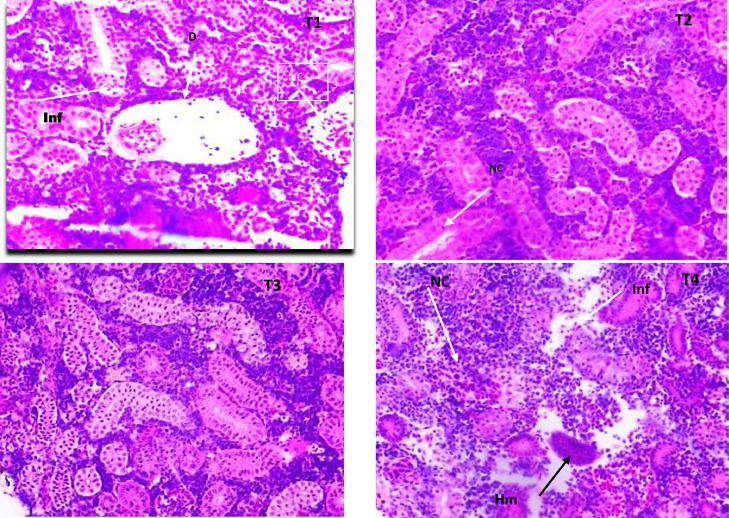
AgNP fed to and challenged by A. hydrophila, resulting in histopathological changes in the kidney of L. rohita (T1:0 µgKg−1, T2:10 µgKg−1, T3:15 µgKg−1, and T4: 20 µgKg−1 AgNP). (H and E stain, x40). Degeneration, necrosis, infiltration, hemosiderin, and NC (Arrow).

**Fig. 8:**
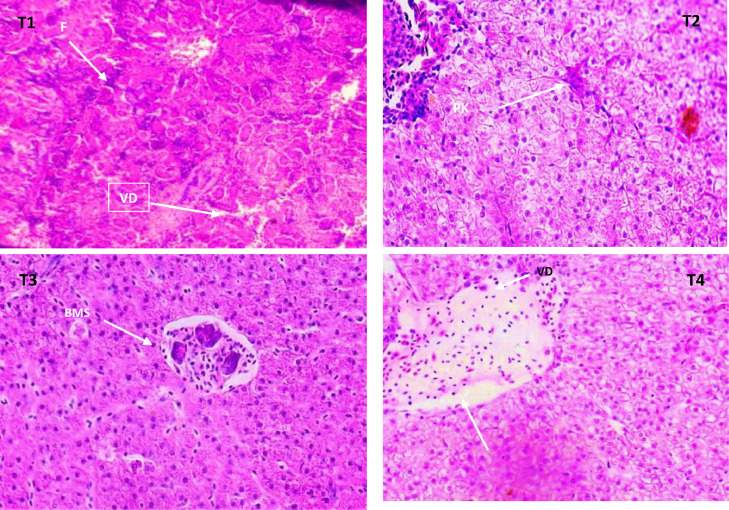
AgNP fed to and challenged by A. hydrophila, resulting in histopathological changes in the liver of L. rohita (T1:0 µgKg−1, T2:10 µgKg−1, T3:15 µgKg−1, T4: 20 µgKg−1 AgNP). (H and E stain, x40). VD = degeneration of the vacuole, PK = pyknosis, F = fibrosis, BMS = Bowman's space.

Fish challenged with *A. hydrophila* after being fed varying amounts of AgNP showed some structural distortion from normal architecture. The branchial arch was widened in the control group, the gill filaments shrunk, and the secondary lamella was lost (Fig. 6a). The gill filament of fish fed with 15 µg AgNP (T3) and *A. hydrophila* challenged exhibited normal appearance, with the exception in gill filaments of T2 (10 g AgNP) that had thickened heads from cellular infiltrations in the sub-epithelium (Fig. 6b). In the control group, higher inclusion level was found to have more severe histological changes. The kidneys of the T4 and control groups both had significant tubular degeneration, as well as interstitial mononuclear cell infiltration. The renal tubules are severed, and the spaces between the tubules and the hemosiderin buildup are invaded by inflammatory cells. After being exposed to *A. hydrophila*, the control group's liver displayed interstitial necrosis, haemorrhages, and inflammatory response (Fig. 7a-d). The hepatic architecture and central veins in the fed fish's liver 10 µg of AgNP appeared to be normal. (Fig. 8b). Higher AgNP concentrations resulted in mild sinusoidal and central vein engorgement, as well as mild inflammatory clusters in the portal region, in the 15 µg AgNP (T3) group (Fig. 8c). In contrast, the 20 g AgNP (T4) group displayed infiltrations close to melanomacrophages, congested hepatic sinusoids, and a few inflammatory cells are present around a swollen portal blood vessel. (Fig. 8d).

### 3.4 Expression of the TGF-β, IL-8, and COX-2, genes

**Fig. 9:**
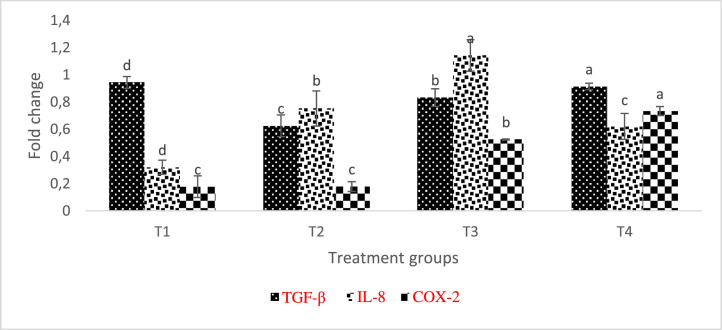
Relative expression of TGF-β, IL-8, and COX-2 transcripts in L. rohita liver fed different inclusion levels of AgNP and later infected with A. hydrophila. The level of transcripts was normalised against -actin. Treatments assigned with matching letters are statistically the same (P < 0.05). (T1:0 µgKg−1, T2:10 µgKg−1, T3:15 µgKg−1 and T4: 20 µgKg−1 AgNP).

The expression of the TGF-β, IL-8, and COX-2, genes in *L. rohita* organs was assessed in response to *A. hydrophila* in comparison to the uninfected group (control). According to AgNP inclusion levels, the studied gene, and the fish organs under investigation, the expression of genes significantly varied (p < 0.05). The fed fish's liver exhibits higher COX-2 and IL-8 expression as AgNP levels rise, but with a sharp decline in IL-8 expression in T4 levels (Fig. 9). TGF- β was decreased in the liver of *L. rohita* fed AgNP diets

**Fig. 10:**
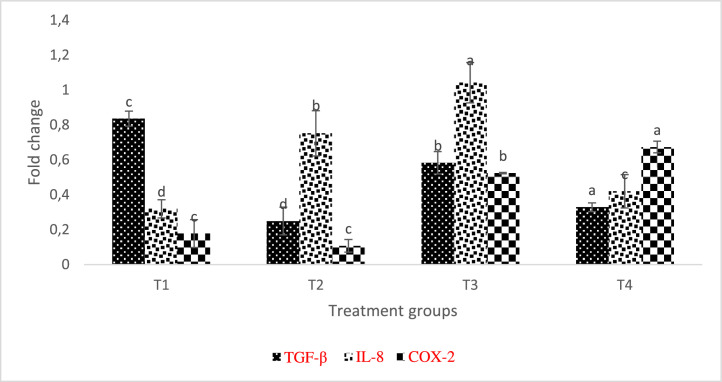
Relative expression of TGF-β, IL-8, and COX-2 transcripts in L. rohita kidney fed different inclusion levels of AgNP and later infected with A. hydrophila. The level of transcripts was normalised against -actin. Treatments assigned with matching letters are significantly the same (P < 0.05). (T1:0 µgKg−1, T2:10 µgKg−1, T3:15 µgKg−1 and T4: 20 µgKg−1 AgNP).

The increased output of IL-8 in the kidneys T2 and T3 was identified by the examined genes' expression patterns. The control group had downregulated levels of COX-2 unlike TGF-β (Fig. 10). Also, COX-2 has significantly higher values in T3 and T4.

**Fig. 11:**
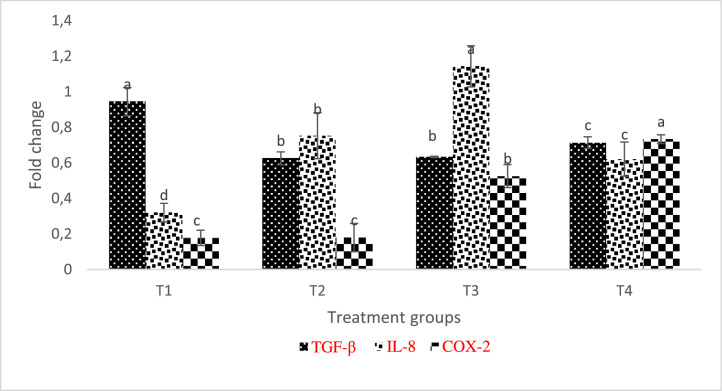
Relative expression of TGF-β, IL-8, and COX-2 transcripts in L. rohita gill fed different inclusion levels of AgNP and later infected with A. hydrophila. The level of transcripts was normalised against -actin. Treatments assigned with matching letters are significantly the same (P < 0.05). (T1:0 µgKg−1, T2:10 µgKg−1, T3:15 µgKg−1 and T4: 20 µgKg−1 AgNP).

**Fig. 12:**
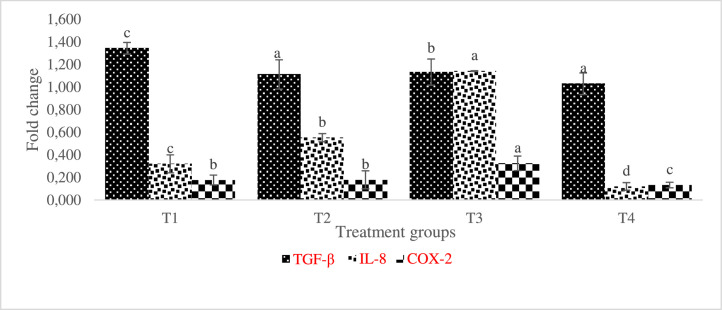
Relative expression of TGF-β, IL-8, and COX-2 transcripts in L. rohita muscle fed different inclusion levels of AgNP and later infected with A. hydrophila. The level of transcripts was normalised against -actin. Treatments assigned with matching letters are statically the same (P < 0.05). (T1:0 µgKg−1, T2:10 µgKg−1, T3:15 µgKg−1 and T4: 20 µgKg−1 AgNP).

COX-2 and IL-8 expression were significantly higher in the gill of AgNP-treated fish with TGF- β expressed upregulation in the control treatment compared with others (Fig. 11). Although IL-8 levels were elevated in T2-T4, they were noticeably higher in T3. IL-8 was statistically higher (p < 0.05) in T2 and T4 when comparing the muscle of the AgNP-fed fish, whereas IL-8 and COX-2 were downregulated in all of the AgNP-included diets except for T3, which had a significantly higher value of COX-2 (Fig. 12)

## 4 Discussion

The most successful approach for preventing fish diseases appears to be enhancing immune responses through the use of nanomaterials as food additives. Their small size and highly specialised surface area give them special characteristics. The percentage of nutrients in fish food that make it through the fish's gut and into its system, as opposed to simply passing through the fish's digestive system unaltered, has been found to increase with the use of nanoparticles in aquafeeds [23,24]. Compared to minerals of a larger size, dietary minerals with a nanoscale size may enter cells more readily, thus, quickening the process of their incorporation into the fish tissue [25]. The addition of mineral nutrients to fish diets regularly at the nanoscale may also have a significant impact on the fish's overall health and growth. In the current study, feeding *L. rohita* a diet supplemented with AgNP up to 15 µgKg^−1^ increased growth performance and feed utilisation, but higher levels resulted in low growth, indicating that AgNP may be toxic at higher dietary levels. This level of inclusion may be appropriate as a dietary mineral supplement that can have a significant impact on an aquatic farm species' effectiveness, absorption, and metabolic pathways [26,27]. Mabrouk et al [28] reported using 10 µg of AgNP for better growth in *O. niloticus*., which is less than the concentrations used in this study, variation in species used and application methods may be responsible for the contrasting values. Fish ingested nutrients from the diet more effectively when AgNP was added to the diet, as evidenced by the decrease in FCR values in the treatment groups. The percentage survival observed in this study confirmed the immunostimulatory property of AgNP. The higher survival percentage in T2 and T3 suggests that the two concentrations could be used to boost immunity in *L. rohita*, but with better performance with 15 µgKg^−1^(T3)

A complex network of many antioxidants, including catalase and SOD, are found in fish [29]. The first line of defense against free radicals is the antioxidant enzymes SOD and CAT, which are important biochemical components. Consequently, measuring these antioxidant parameters could indicate the fish's antioxidant capacity and act as biomarkers for oxidative stress [30]. The presence of stressors has been reported to increase oxidative stress as measured by catalase, SOD, and GST [31,29]. Additionally, Kumar [32] demonstrated that increased oxidative stress is seen in natural water bodies when there is an infection. Reduced catalase and SOD activity and elevated NBT levels show that AgNP supplementation at the specified doses (10 and 15 g/Kg) alleviated oxidative stress in all investigated tissues. Additionally, the 20 µg/Kg concentration of AgNP causes an increase in catalase activity, which is a sign of oxidative stress. Nevertheless, it is confirmed that at higher quantities of AgNP, Ag+, which could be obstructed by sulphydryl reagents of mitochondria, causes toxicity to the tissue [33]. In a similar vein, AgNP has been reported to significantly decrease antioxidative enzymes in fish [34]. Moreover, oxidative damage was reduced at lower AgNP concentrations (10 and 15 µg/kg), so these optimal levels act as stress mitigators. The concentrations of AgNP (10 µg/Kg diet) supplementation influenced L. rohita's immunity in terms of NBT. The intracellular superoxide radicals generated by leucocytes were used to calculate the NBT value, which represents the respiratory burst activity of phagocytes. Recent studies suggested that AgNP also triggers an immune response in biological hosts, involving immune cells [35]. According to the current study, AgNP delivered through diet operates as an immunomodulator at low concentrations while acting as an immune inhibitor at higher concentrations (20 µg/Kg diet) in *L. rohita*.

In the control group (normal diet), the histology of the liver revealed aberrant hepatocytes, whereas the treated group's liver tissue had mild to severe changes. The abnormality observed in the control might be the effect of the *A. hydrophila* and that the immune status was not improved unlike AgNP supplemented group. The severe alterations in liver tissue observed at a higher level of AgNP (T4) might be linked to the toxicity of AgNP. Rajkumar [36] showed that fish treated with AgNP had a congestive expansion of liposomes, which caused vacuolar degeneration in the liver. There was more necrosis than was seen in T4 within the fish liver tissues fed to AgNP. Certainly, among the most noticeable effects of a toxin on tissues is necrosis [37,38]. Necrosis can also be caused by enzyme inhibition, disruption of the cellular membrane, issues with protein synthesis, and issues with gluconeogenesis [39]. The level of xenobiotics present during the detoxification process is also related to the presence of the necrosis region in fish liver. [37] linked the high number of neutrophils in the kidneys of those given AgNP to the release of tumoral factors by local macrophages within the damaged tissues. Fish fed with inorganic contaminants had neutrophilic infiltration, according to Hinton [40].

In terms of the gill, modifications such as widening of the branchial arch, reduction of the gill filaments, and disappearance of secondary lamella were seen in the reference and higher inclusion levels. The *A. hydrophila* infection possibly contributed to the modifications, even more so in the absence of immunostimulants in the control group and/or the toxicity of AgNP in the T4. Fish mortality due to structural gill destruction has been documented [41], but the majority of the reports concentrated on bathing or immersion rather than dietary inclusion. However, earlier aquatic organism research discovered AgNP buildup in the tissues [42,43]. By interfering with the H+, Cl-, and Na+ transfers at the gill surface, silver nanoparticles tend to cause cardiovascular collapse and impair the gills' ability to carry out their normal functions. However, the precise mechanism of action for kidney toxicity and renal alterations caused by silver nanoparticles is unknown [44].

Understanding the fundamental workings of immunity and identifying new strategies for better management in the aquaculture sector requires an understanding of the biosynthesis of immune molecules and their responses to pathogens. The aquaculture of Indian big carp has had a significant setback in recent years as a result of related disease concerns, including infections brought on by *A. hydrophila* [45]. Our research shows that, in addition to improving immunological response, diet enrichment with AgNPs can successfully control the expression of specific genes in *L. rohita*. In the current investigation, after *L. rohita* was challenged with *A. hydrophila*, IL-8 and COX-2 levels were elevated in the gill and liver of AgNP-enriched diets supplied to the fish in contrast to the control.

Inflammation-related IL-8 is a significant protein that is essential for attracting neutrophils and other immune cells to the site of infection [46]. Epithelial, endothelial, and airway smooth muscle cells are additional sources of IL-8 in addition to macrophages. It has been demonstrated that this chemokine plays a role in a variety of cellular processes, including angiogenesis, tissue remodeling, and cell proliferation [47]

There was a noticeable increase in many fish species in IL-8 expression in reaction to different stressors [29,48]. After being induced with LPS, rainbow trout larvae express IL-8, indicating that it significantly contributes to early immune system stages [49] TGF- β, which is defined by its ability to moderate the immune system, prevents B and T cells from multiplying, and differentiating alongside combats inflammation-promoting cytokines [50]. In this study, AgNP-fed, *A. hydrophila* -infected *L. rohita* showed decreased relative TGF-mRNA expression in the kidney, muscle, liver, and gill.

Given that it is a cytokine with regulatory anti-inflammatory properties, its downregulation in this study may have contributed to the upregulation of the transcription of additional pro-inflammatory genes.

The observed increase in COX-2 expression, which is an inductive enzyme present in the inflammatory stimulus, could be the result of inflammation in the liver, kidney, and gills which might be a result of disturbs by the presence of nanosilver in the kidney, gill and liver tissues, especially at high inclusion level. T4 group (the highest dose of silver) fish presented higher COX-2 expression, but these results do not influence the fish performance. This might probably be because the AgNPs treatment controlled specific groups of microorganisms in the gut, favoring the performance, but its excess could have damaged the mucosa.

Furthermore, Devi et al [51] observed that young European sea bass, *Dicentrarchus labrax*, fed a mannan-oligosaccharide diet had lower TGF-β expression in the gut. Additionally, Torrecillas et al [52] found that *L. rohita* leucocytes on a diet supplemented with galactooligosaccharides showed a decrease in the expression of the TGF-β gene.

As opposed to this, Qin [53] discovered that tilapia fed a diet rich in chitooligosaccharides had increased transcription rates of TGF- β in their intestines. These conflicting results are intriguing because they highlight the particular influence of nano additives, particularly AgNP and the necessary dose, on the control of immunomodulation parameters. Modulating the aforementioned immune-responsive genes in infected *L. rohita* that have been fed AgNP may be crucial to combating *A. hydrophila* pathogenic mechanisms and improving survival rates.

The results of this experiment indicate that AgNP, when used at the recommended level of 15 g/Kg, has superior antibacterial activity against *A. hydrophila.* Additionally, the growth performance, immunity, and antioxidant status of the fish-fed diets containing AgNP (10 and 15 g/Kg) improved as did the expression of immune genes. However, histological abnormalities were present at the maximum (20 g/Kg) AgNP concentration, suggesting that this level of AgNP may be harmful to cultured fish. The research findings demonstrated that a diet containing AgNP at a dose of 15 µg/Kg or less both strengthens L. rohita's innate immune system and antioxidant capability, with any dose over that (15µg/Kg) potentially harmful to the fish. The results suggest that AgNP may be an effective alternative to antibiotics and vaccines for the prevention of pathogenic bacteria in aquaculture.

## Author's contributions

The experiments were developed and designed by Omoniyi Michael Popoola, who also obtained and evaluated the data, and drafted the report. Additionally, he provided materials and tools for analysis. Bijay Kumar Behera and Vikash Kumar supplied chemicals, equipment, and wares in addition to conceptualizing and designing the experiments, carrying them out, and editing the report.

## Declaration of Competing Interest

No competing interests are disclosed by the authors.

## Data Availability

No data was used for the research described in the article.
